# Author Correction: Dynamical nonlinear memory capacitance in biomimetic membranes

**DOI:** 10.1038/s41467-019-11779-5

**Published:** 2019-08-21

**Authors:** Joseph S. Najem, Md Sakib Hasan, R. Stanley Williams, Ryan J. Weiss, Garrett S. Rose, Graham J. Taylor, Stephen A. Sarles, C. Patrick Collier

**Affiliations:** 1 Department of Mechanical, Aerospace and Biomedical Engineering, University of Tennessee, Knoxville, TN 37916 USA; 2 Joint Institute for Biological Sciences, Oak Ridge National Laboratory, Oak Ridge, TN 37831 USA; 3 Department of Electrical Engineering and Computer Science, University of Tennessee, Knoxville, TN 37916 USA; 4 Department of Electrical and Computer Engineering, Texas A&M University, College Station, TX 77840 USA; 5 Bredesen Center for Interdisciplinary Research, University of Tennessee, Knoxville, TN 37996 USA; 6 Center for Nanophase Materials Sciences, Oak Ridge National Laboratory, Oak Ridge, TN 37831 USA

**Keywords:** Membrane biophysics, Electrical and electronic engineering, Self-assembly, Nanoscale devices

Correction to: *Nature Communications* 10.1038/s41467-019-11223-8, published online 19 July 2019

The original version of this Article contained an error in Fig. 3b, in which the x-axis label incorrectly read ‘Time (s)’ rather than the correct ‘*V* (V)’. This has been corrected in both the PDF and HTML versions of the Article.

