# Supplementary material for: Dynamical nonlinear memory capacitance in biomimetic membranes
Source: Nat Commun. 2019 Jul 19;10:3239. doi: 10.1038/s41467-019-11223-8 (PMC6642212; doi:10.1038/s41467-019-11223-8)
Supplement: Supplementary file 1 — Supplementary Information [file 41467_2019_11223_MOESM1_ESM.pdf]

Supplementary Information

Dynamical nonlinear memory capacitance in biomimetic membranes

Najem *et al.*

Supplementary Note 1: Amphiphilic Nature of Phospholipids

The plasma membranes of living cells (e.g., neurons) are made of lipid bilayers (~50% lipids by mass[1]) that also contain other molecules, including carbohydrates, sterols, and many types of proteins[2]. Phospholipids, being the most abundant types of membrane lipids, are known to be amphipathic—that is, possess a hydrophilic (water-loving) phosphate headgroup and two hydrophobic (water-hating) acyl chains (Supplementary Figure 1b). The basic structure of a cell membrane is a lipid bilayer in which the polar headgroups orient toward the aqueous exterior and the acyl chains point inward[2] (Supplementary Figure 1). Lipid bilayers are largely impermeable to ions and polar molecules—allowing cells to regulate the movement of these substances via transmembrane protein complexes, such as pores and channels. Inspired by this arrangement, and taking advantage of the intrinsic amphipathic nature of phospholipids, we assemble planar lipid bilayer membranes between two lipid-coated water droplets in oil using DPhPC synthetic phospholipids as described in Fig. 1.

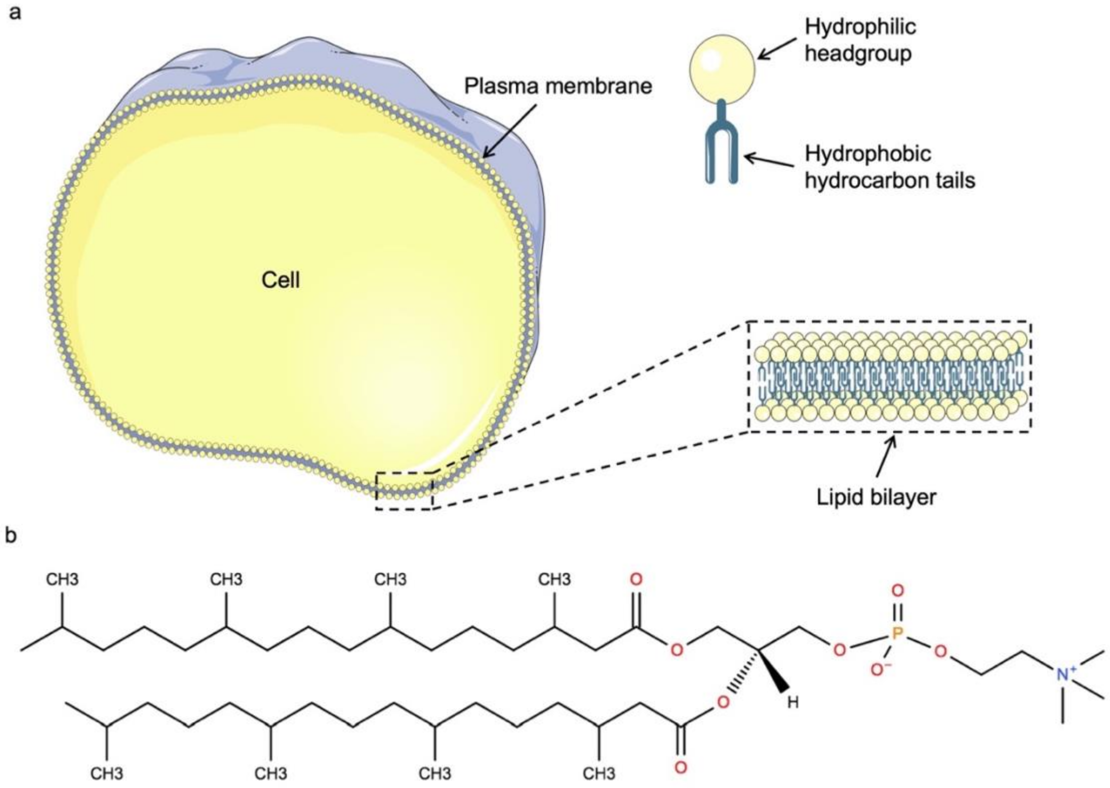

Supplementary Figure 1. Schematics showing a cell, the plasma membrane, and a phospholipid. a, The plasma membrane forms a diffusive barrier between the inside of a cell and its extracellular surroundings. The plasma membrane allows selective transport and signalling through a variety of membrane proteins embedded in the lipid bilayer. The amphipathic nature of phospholipids allows their self-assembly at an oil-water interface needed for the formation of planar lipid bilayers between droplets as described in Fig. 1. **b,** The chemical structure of the DPhPC phospholipid used throughout this study.

Note: The graphics used in this Supplementary Figure 1 were modified from Servier Medical Art (http://smart.servier.com), licensed under a Creative Common Attribution 3.0 Generic License (https://creativecommons.org/licenses/by/3.0/).

Supplementary Note 2: Equivalent Electrical Circuit and Specific Capacitance of a Lipid Bilayer

As described in the text, a lipid bilayer is modelled as a parallel-plate capacitor wired in parallel with a high ohmic resistance (i.e., > 10 GΩ). The high dielectric constants (~80) of the polar headgroups on both sides of the bilayer means that for capacitors in series, the low dielectric (~2-3) hydrophobic core of the membrane is the dominate source of capacitance at the interface[3]. Because the planar membrane formed between pendant droplets is elliptical (major axis oriented in the vertical direction[4]) with a minor axis radius, R, and hydrophobic thickness, W, the nominal capacitance, C, is expressed as follows,

$$C = \frac{\varepsilon\varepsilon_0 a\pi R^2}{W}, \tag{S.1}$$

where, ε is the relative dielectric constant of the hydrophobic core of the lipid membrane, ε_0, is the permittivity of free space (8.854×10^{-12} F m^{-2}), and a is the ellipticity factor ($a = R_{\mathrm{major}}/R$) that accounts for sagging of aqueous droplets hanging on electrodes in the oil. Specific capacitance, C_m, for a membrane is obtained by normalizing the nominal capacitance with respect to bilayer area:

$$C_{\mathrm{m}} = \frac{C}{A_{\mathrm{b}}} = \frac{\varepsilon\varepsilon_0}{W}. \tag{S.2}$$

Since ε_0 is a physical constant and ε is fixed for a given lipid composition, knowledge of C_m provides direct information on W. For a precise experimental determination of C_{m} for droplet interface bilayers, one can refer to Taylor *et al*[4].

Supplementary Note 3: Assembly of a Droplet Interface Bilayer

The hydrophilic headgroups of phospholipids are readily solvated by water, while the hydrophobic tails are insoluble in an aqueous environment. Therefore, if phospholipids such as DPhPC are dispersed in a water droplet placed in oil, they self-assemble within a few minutes to form a well-packed lipid monolayer to reduce the interfacial energy at the water/oil interface (Fig. 1a). The tension of the water-hexadecane interface is ~44 mN m^{-1} in the absence of lipids, whereas a well-packed DPhPC monolayer lowers this value[5] to ~1.18 mN m^{-1}.

When two lipid-covered droplets are brought into contact (Fig. 1a and Supplementary Figure 2), the lipid tails from each droplet interact with each other at the interface to expel excess oil and form an oil-depleted hydrophobic centre (i.e., a thinned lipid bilayer), while the headgroups in each bilayer leaflet interact with each other to form a hydrophilic boundary on each side of the bilayer (Fig. 1). This process occurs spontaneously when the oil is a relatively “poor” solvent for the lipid tails, for which tail-tail interactions are more favourable than tail-oil interactions[6]. Exclusion of oil from between opposing lipid leaflets is an entropically favoured process (i.e., the oil can maximize its entropy by not remaining confined in the membrane) that results in a thinned bilayer stabilized by steric forces exerted by opposing lipid tails.

As described in Methods, we use a hanging-drop technique described elsewhere[4,7] to assemble the bilayers. Supplementary Figure 2 shows bottom view images that illustrate steps in the assembly process, namely before bringing droplets into contact (A), after bringing droplets into contact (B), and after lipid bilayer formation C (see Supplementary Movies 1-3 for real-time display of lipid bilayer formation).

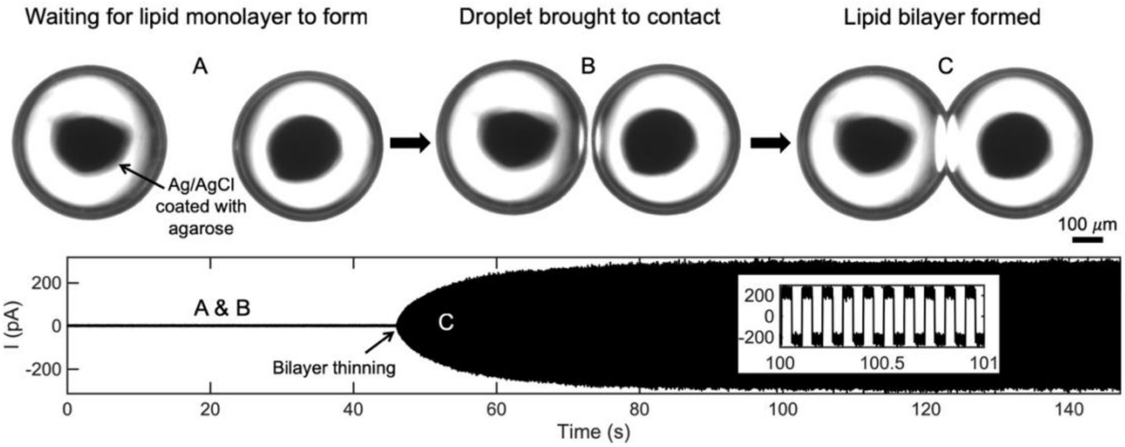

Supplementary Figure 2. Bottom-view micrographs showing the assembly process of a lipid bilayer between two lipid-coated aqueous droplets in oil. After depositing 200 nL of lipid solution on each of the Ag/AgCl wires under oil, the droplets incubate for 5 minutes to allow sufficient lipid monolayer formation at the water/oil interface (A). Droplets are then placed in contact by repositioning the electrodes via micromanipulators (B); and, within a minute, the bilayer starts forming (i.e., the area of thinned membrane grows steadily). The bilayer interface typically reaches a steady-state area of contact within ~ 20 seconds (C). Bilayer formation is reflected as an increase in the amplitude of square waveform capacitive current induced by a 10 Hz, 10 mV triangular voltage. For states A and B, the capacitive current is <10 pA. However, after bilayer formation, the peak-to-peak capacitive current is ~ 500 pA at steady state.

Supplementary Note 4: Equilibrium Geometry of a Droplet Interface Bilayer

Upon bilayer formation, the adhesive interface reaches mechanical equilibrium via balance of interfacial tensions described by Young's equation[4]:

$$\gamma_{\mathrm{b}} = 2\gamma_{\mathrm{m}} \cos\frac{\theta_{\mathrm{b}}}{2}. \tag{S.3}$$

According to Eq. S.3, bilayer tension, γ_{b}, is balanced by the tangential components of the two monolayer tensions, γ_{m}, while the opposing normal components of γ_{m} add to zero. At zero membrane voltage, bilayer tension, $\gamma_{\mathrm{b,0}}$ and monolayer tension, γ_{m}, find equilibrium at a droplet contact angle, θ_0. For γ_{m} = 1.18 mN m^{-1} and θ_0 = 30°,[4] the zero-volt bilayer tension, $\gamma_{\mathrm{b,0}}$, is ~ 2 mN m^{-1}. Monolayer tensions and contact angles are directly dependent on the type of oil used. Supplementary Movies 1-3 display, in real-time, the formation of a DPhPC bilayer in decane, hexadecane, and squalene, respectively. One can observe in these videos that bilayer area, at zero membrane potential, is the smallest in the case of decane and the largest in the case of squalene. As explained by Taylor *et al.*[4], the initial size of the lipid bilayer is defined by the available specific free energy of bilayer adhesion (0.078 mN m^{-1}, 0.302 mN m^{-1}, and ~ 2 mN m^{-1} for decane[4], hexadecane[4], and squalene (estimated), respectively).

An applied voltage, *v*, reduces the bilayer tension via electrowetting (EW), as given by the following form of the Young-Lippmann[4] relationship:

$$\Delta\gamma_{\mathrm{b}} = 2\gamma_{\mathrm{m}}\left(\cos\frac{\theta_{\mathrm{b}}'}{2} - \cos\frac{\theta_{\mathrm{b}}}{2}\right) = -\frac{C_{\mathrm{m}}}{2}v^2, \tag{S.4}$$

where C_{m} is the specific capacitance of the membrane. Since γ_{m} is unaffected by voltage, the reduction in γ_{b} ($\gamma_{\mathrm{b}}' < \gamma_{\mathrm{b,0}}$) causes the angle of the bilayer under voltage to grow from its zero-

volt value ($\theta_b' > \theta_b$). Coupled to fixed droplet volumes, this increase in contact angle increases the radius of contact ($R_v > R_0$) to maintain adhesive equilibrium.

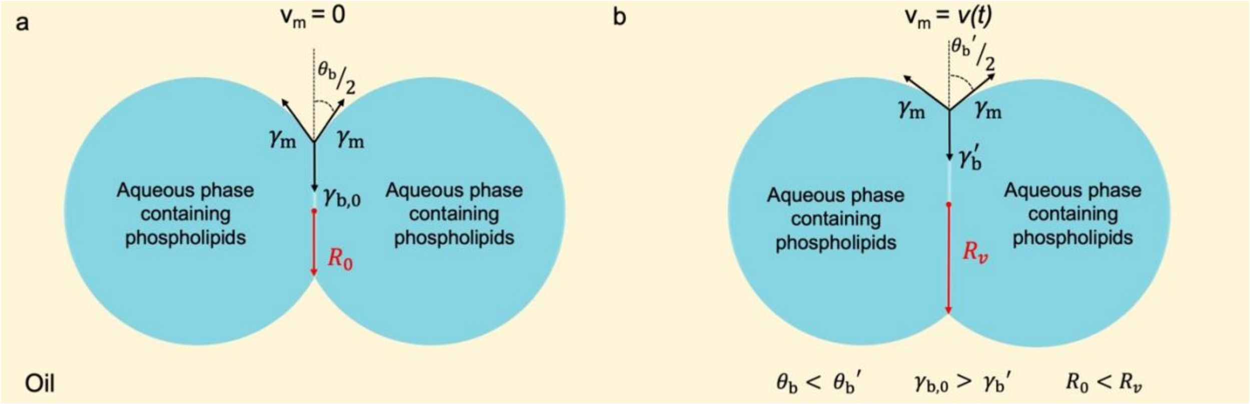

Supplementary Figure 3. Schematic (bottom view) showing adhesion equilibrium in the absence and presence of a nonzero membrane voltage. a, The zero-volt bilayer tension, $\gamma_{b,0}$, is equal to the sum of the vertical components of the two opposing monolayer tensions, γ_m, as determined by the zero-volt contact angle, θ_b. The red arrow indicates the equilibrium value of the minor axis radius, R_0, at zero voltage bias. **b,** Through EW at constant thickness, the application of a voltage bias, $v(t)$, leads to a decrease in bilayer tension, γ_b', and an increase in bilayer contact angle, θ_b', that increases the radius of the membrane. In addition, $v(t)$ may also lower W via electrocompression (EC), a change that couples implicitly to the EW response by altering the value of C_m in Eq. S.4.

Supplementary Note 5: Empirical Quasi-static Responses to an Applied Potential

As described above and in the text, a non-zero membrane voltage leads to an increase in the droplet contact angle, θ_b, through EW, which results in the formation of new membrane area between droplets. We[4,8] and others[9] have found that bilayer area, A_b, increases reversibly and quadratically with the magnitude of the applied voltage, v, as given by

$$A_\mathrm{b}(v) = A_{\mathrm{b},0}(1 + \beta v^2), \qquad \text{(S.5)}$$

where $A_{\mathrm{b},0}$ is the area of the lipid bilayer at zero volts and β is the quasi-static fractional increase in bilayer area per voltage squared. Supplementary Figure 4 compares the area sensitivity to voltage for DPhPC membranes in hexadecane ($\beta \sim 14.5\, V^{-2}$) and decane ($\beta \sim 60\, V^{-2}$). In a similar manner, EC causes C_m to increase quadratically with voltage, as given by

$$C_\mathrm{m}(v) = C_{\mathrm{m},0}(1 + Bv^2), \qquad \text{(S.6)}$$

where $C_{\mathrm{m},0}$ is the specific capacitance at zero volts and B is the quasi-static fractional increase in C_m. Supplementary Figure 4 also displays estimates of B for DPhPC bilayers in hexadecane ($B \sim 0.5\, V^{-2}$) and decane ($B \sim 12.3\, V^{-2}$). These data show that while DPhPC bilayers in decane exhibit significant increases in area and specific capacitance (i.e. decreases in thickness) due to EW and EC, respectively, those in hexadecane exhibit increases in area at nearly constant thickness (i.e., negligible EC).

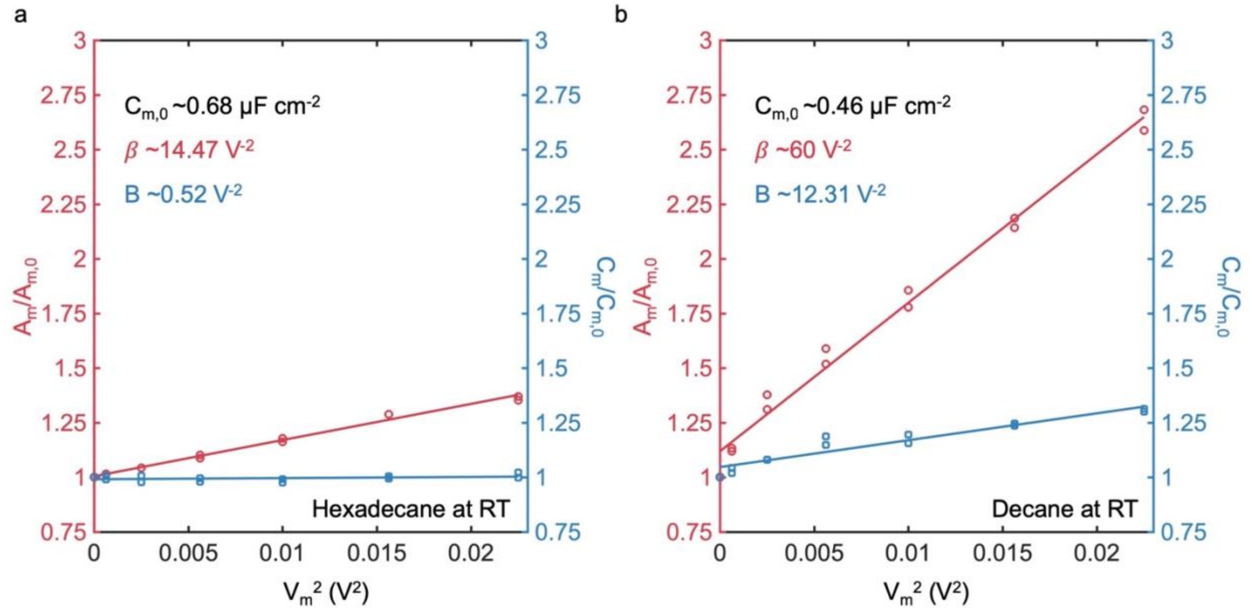

Supplementary Figure 4. Normalized quasi-static changes in membrane area and specific capacitance versus voltage squared for DPhPC membranes in hexadecane and decane. A DPhPC membrane exhibits a larger, zero-volt specific capacitance value when formed in hexadecane (0.68 μF cm^{-2}) as opposed to decane (0.46 μF cm^{-2}). These values indicate that DPhPC membranes formed in decane are thicker (~48%) than those formed in hexadecane. These data also highlight that a DPhPC bilayer in decane experiences greater fractional increases in area and specific capacitance compared to one in hexadecane.

To explore the voltage dependence of capacitance, we recall that nominal capacitance, C, is the product of bilayer area and specific capacitance, as given by:

$$C = A_{\mathrm{b}} C_{\mathrm{m}} \tag{S.7}$$

Thus, Eqs. S.5-7 can be combined to write capacitance as a function of voltage:

$$C(v) = A_{\mathrm{b},0} C_{\mathrm{m},0} (1 + (\beta + B) v^2 + \beta B v^4). \tag{S.8}$$

Equation S. 8 shows that quasi-static capacitance is a quadratic function of voltage for appreciably small voltages (< 0.2 V) and when either β or B are small. However, when β, B, and v are not small, nominal capacitance is a fourth-order function of voltage. This variable order (and sensitivity to voltage) affects the shape of the $Q - v$ relationship, since $Q = Cv$.

Supplementary Note 6: Mathematical Derivations of Memcapacitance State Equations

As explained in the manuscript, a non-zero membrane potential, $v_{app}(t)$ across the memcapacitor can: 1) increase the area of the interface due to EW, and 2) reduce the membrane thickness due to EC. Both phenomena are expected to be hysteretic due to the presence of time constants that create inherent time lags. Therefore, the system's state variables are chosen to be the minor axis radius, *R*, (which is directly measurable in the experimental setup) and the hydrophobic thickness, *W*.

The charge, Q, across the memcapacitor is given by,

$$q(v,t) = C\big(R(v,t), W(v,t)\big)v(t), \tag{S.9}$$

where memcapacitance, *C*, is given by

$$C(R,W) = \frac{\varepsilon\varepsilon_0 a\pi\big(R(t)\big)^2}{W(t)}. \tag{S.10}$$

Based on the free-body diagram in Supplementary Figure 5 and knowledge of first-order dynamics for changes in radius and thickness, we write the equations of motion for EW and EC as follows,

$$\xi_{\mathrm{ew}}\frac{dR(t)}{dt} + k_{\mathrm{ew}}(R(t) - R_0) = f_{\mathrm{ew}}(v,t) \tag{S.11}$$

and

$$\xi_{\mathrm{ec}}\frac{dW(t)}{dt} + k_{\mathrm{ec}}(W(t) - W_0) = -f_{\mathrm{ec}}(v,t), \tag{S.12}$$

where ξ_{ew} and k_{ew} are the effective damping (N s m^{-2}) and stiffness (N m^{-2}) coefficients, respectively, in the tangential direction, ξ_{ec}, and k_{ec} are the effective damping (N s m^{-1}) and stiffness (N m^{-1}) coefficients, respectively, in the normal directions, and f_{ew} and f_{ec} are the effective

EW tension and EC force, respectively, resulting from the applied voltage. Both equations of motion are written with respect to the equilibrium states, R_0, and W_0, at zero volts.

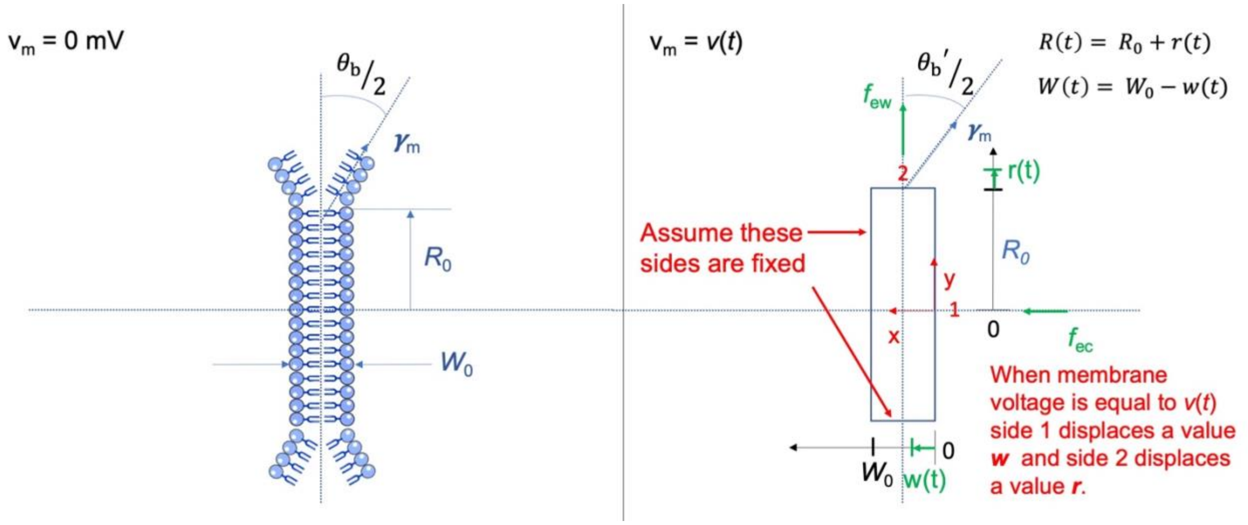

Supplementary Figure 5. A schematic illustrating the EW and EC forces acting on the membrane in the presence of a voltage bias. The application of a bias voltage will decrease the tension in the bilayer, which will drive an increase in the bilayer area at constant thickness. This decrease in bilayer tension is equivalent to f_{ew} acting in the positive direction as the voltage bias increases. In addition, a perpendicular force, f_{ec}, acts on the membrane, in the presence of a bias—reducing its thickness (i.e., a positive f_{ec} results in a negative change in W).

Supplementary Note 7: Mathematical derivations of EW and EC forces

Energy derivations

Let's consider a circuit consisting of a battery and a memcapacitor. The supplied energy, E_{sup}, could be defined as a function of applied voltage, *v*, and current, *i*, as follows,

$$E_{sup} = \int_0^t vidt. \tag{S.13}$$

Knowing that $idt = dq$, Eq. S.13 becomes,

$$E_{\text{sup}} = \int_0^q vdq, \tag{S.14}$$

which can also be written as,

$$E_{\text{sup}} = \int_0^v vCdv + \int_0^C v^2 dC. \tag{S.15}$$

Using the product rule of integration, we get,

$$E_{\text{sup}} = \frac{Cv^2}{2} - \frac{C_0 v(0)^2}{2} - \frac{1}{2}\int_{C_0}^{C} v^2 dC + \int_{C_0}^{C} v^2 dC \tag{S.16}$$

which becomes

$$E_{\text{sup}} = \frac{Cv^2}{2} - \frac{C_0 v(0)^2}{2} + \frac{1}{2}\int_{C_0}^{C} v^2 dC , \tag{S.17}$$

where C_0 is the membrane capacitance at zero membrane potential. Our derivations reveal that the term $\frac{1}{2}\int_0^C v^2 dC$ is the capacitive memory term, which shows the effect of the dynamical change in capacitance—in the case of a static capacitor, this term is zero. Therefore, the supplied energy is used for: 1) capacitive energy storage and 2) energy dissipation to drive the changes in capacitance (i.e., memory term).

If the initial stored energy in the voltage source is E_0 and energy stored in the capacitor is E_c, the total energy in the system, E_{sys} becomes

$$E_{\mathrm{sys}} = E_0 - E_{\mathrm{sup}} + E_{\mathrm{c}}. \tag{S.18}$$

Replacing each term by its expression leads to,

$$E_{\mathrm{sys}} = E_0 + \frac{C_0 v(0)^2}{2} - \frac{1}{2}\int_{C_0}^{C} v^2 dC \tag{S.19}$$

Electrowetting tension

The effective electrowetting tension, f_{ew}, can be obtained by differentiating the system's energy, E_{sys}, with respect to bilayer area as follows,

$$f_{\mathrm{ew}} = -\frac{\partial E_{\mathrm{sys}}}{\partial A_{\mathrm{b}}}. \tag{S.20}$$

Replacing E_{sys} with Eq. S.19 gives us

$$f_{\mathrm{ew}} = -\frac{\partial\left(E_0 + \frac{C_0 v(0)^2}{2} - \frac{1}{2}\int_{C_0}^{C} v^2 dC\right)}{\partial A_{\mathrm{b}}}. \tag{S.21}$$

Knowing that the first two terms of E_{sys} are constants, Eq. S.21 becomes

$$f_{\mathrm{ew}} = \frac{1}{2}\frac{\partial\left(\int_{C_0}^{C} v^2 dC\right)}{\partial A_{\mathrm{b}}}. \tag{S.22}$$

Differentiating E_{sys} with respect to bilayer area (Eq. S.20) leads to

$$f_{\mathrm{ew}} = \frac{a\varepsilon\varepsilon_0}{2W(t)} v(t)^2. \tag{S.23}$$

Combining Eqs. S.11 and S.21, the state equation for R due to EW becomes,

$$\frac{dR(t)}{dt} = \frac{1}{\xi_{\mathrm{ew}}}\left(\frac{a\varepsilon\varepsilon_0}{2W(t)}v(t)^2 - k_{\mathrm{ew}}(R(t) - R_0)\right). \tag{S.24}$$

Electrocompression force

Similarly, the electrocompressive force, f_{ec}, is obtained by differentiating the negative of the system's energy, E_{sys}, with respect to bilayer thickness, as follows,

$$f_{\mathrm{ec}} = -\frac{\partial E_{\mathrm{sys}}}{\partial w} = \frac{\partial E_{\mathrm{sys}}}{\partial W}. \tag{S.25}$$

Differentiating the system's energy with respect to W leads to,

$$f_{\mathrm{ec}} = \frac{\varepsilon\varepsilon_0 a\pi R(t)^2}{2W(t)^2}v(t)^2. \tag{S.26}$$

Combining Eqs. S12 and S.25, the state equation for W due to EC becomes,

$$\frac{dW(t)}{dt} = \frac{1}{\xi_{ec}}\left(-\frac{a\pi\big(R(t)\big)^2}{2\big(W(t)\big)^2}\varepsilon\varepsilon_0 v(t)^2 + k_{\mathrm{ec}}\big(W_0 - W(t)\big)\right). \tag{S.27}$$

From experiments, we know that W cannot be less than a certain minimum width, W_{min} ~2.2 nm. To avoid unphysical values of W, we used a conditional in MATLAB implementations to ensure that W never goes below W_{min}.

We defined the conditional as follows:

if $(W > W_t)$:

$$\frac{dW(t)}{dt} = \frac{1}{\xi_{\mathrm{ec}}}\left(-\frac{a\pi\big(R(t)\big)^2}{2\big(W(t)\big)^2}\varepsilon\varepsilon_0 v(t)^2 + k_{\mathrm{ec}}\big(W_0 - W(t)\big)\right) \tag{S.28}$$

else:

$$\frac{dW(t)}{dt} = \frac{1}{\xi_{\mathrm{ec}}}\left(-\frac{a\pi\big(R(t)\big)^2}{2\big(W(t)\big)^2}\varepsilon\varepsilon_0 v(t)^2\left(\frac{W(t)-W_{\mathrm{min}}}{W_{\mathrm{t}}-W_{\mathrm{min}}}\right)+k_{\mathrm{ec}}\big(W_0-W(t)\big)\right). \tag{S.29}$$

This equation preserves the continuity of $\frac{dW(t)}{dt}$ and ensures that $\frac{dW(t)}{dt} \to 0$ as $\boldsymbol{W}(t) \to W_{\mathrm{min}}$.

We chose $W_t = W_{min} + 0.1$ (*nm*).

Supplementary Methods: Fitting Routine and Simulations Results

We used a non-linear least squares data fitting routine (*lsqcurvefit* in MATLAB) to estimate values for the four parameters ($\xi_{\mathrm{ew}}, k_{\mathrm{ew}}, \xi_{\mathrm{ec}}, k_{\mathrm{ec}}$) by simultaneously fitting independent, representative measurements of capacitance, *C*, and radius, *R*, to Eqs. S.24 and S.27. These routines were performed separately for data obtained on membranes in hexadecane and decane as described below.

Hexadecane

Based on the experimental evidence we presented in both the manuscript and in the SI, we know that a DPhPC bilayer in hexadecane exhibits nearly constant thickness at voltages of 150 mV and less. Therefore, we assumed variations in capacitor geometry are fully predicted by a modified form of Eq. S.24 given by

$$\frac{dR(t)}{dt} = \frac{1}{\xi_{\mathrm{ew}}}\left(\frac{a\pi}{2W_0}\varepsilon\varepsilon_0 v(t)^2 - k_{\mathrm{ew}}(R(t) - R_0)\right). \qquad \text{(S.30)}$$

In this case, where $W = W_0$, a closed form analytical solution for $R(t)$ to both a pulse and sinusoidal inputs is known.

Decane

Based on the quasi-static responses in area and specific capacitance shown in Fig. S4, we know that in decane both EW and EC contribute to changes in capacitance. Therefore, we used the implicitly coupled state equations given by S.25 and S.27. We used MATLAB lsqcurvefit, a non-linear least square data fitting routine, to find the four parameters, ζ_{ew}, k_{ew}, ζ_{ec}, k_{ec}, by fitting simultaneous experimental measurements of capacitance, *C*, and radius, *R*, to the system equations. We found that the fitted parameters changed with the frequency of the applied voltage. Moreover,

we found that the damping and stiffness parameters may not be constant during the initial transient since our system is not a closed-volume system.

Metrics for goodness of fit

In addition to qualitative assessment through visual inspection, we have used two metrics, namely MAPE (Mean Absolute Percentage Error) and R^2 (Coefficient of Determination) to evaluate the goodness of fit.

MAPE is defined as follows,

$$MAPE = \left(\frac{1}{n}\sum_{i=1}^{n}\left|\frac{E_i - M_i}{E_i}\right|\right) \times 100\%, \qquad \text{(S.31)}$$

where E_i is the experimental value and M_i is the model output.

R^2 was calculated for each case as follows,

$$R^2 = 1 - \frac{\sum_{i=1}^{n}(E_i - M_i)^2}{\sum_{i=1}^{n}(E_i - \bar{E})^2}. \qquad \text{(S.32)}$$

The damping and stiffness parameters of the model have been determined using a fitting routine with a 95% confidence interval (±1.96 σ). The confidence interval along with MAPE and R^2 are shown in Table 1.

Supplementary Figures:

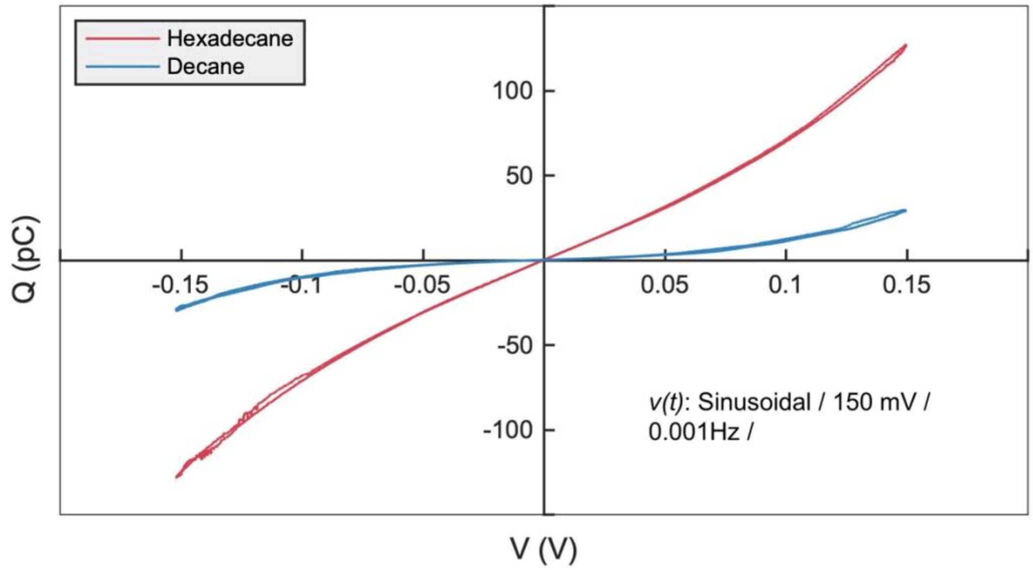

Supplementary Figure 6. Response of the system to low-frequency sinusoidal voltage bias. Bilayers in both oils exhibit nonlinear, nonhysteretic *Q*-*v* relationships at very low frequencies. These are quasi-DC measurements in which the system is essentially always at steady state.

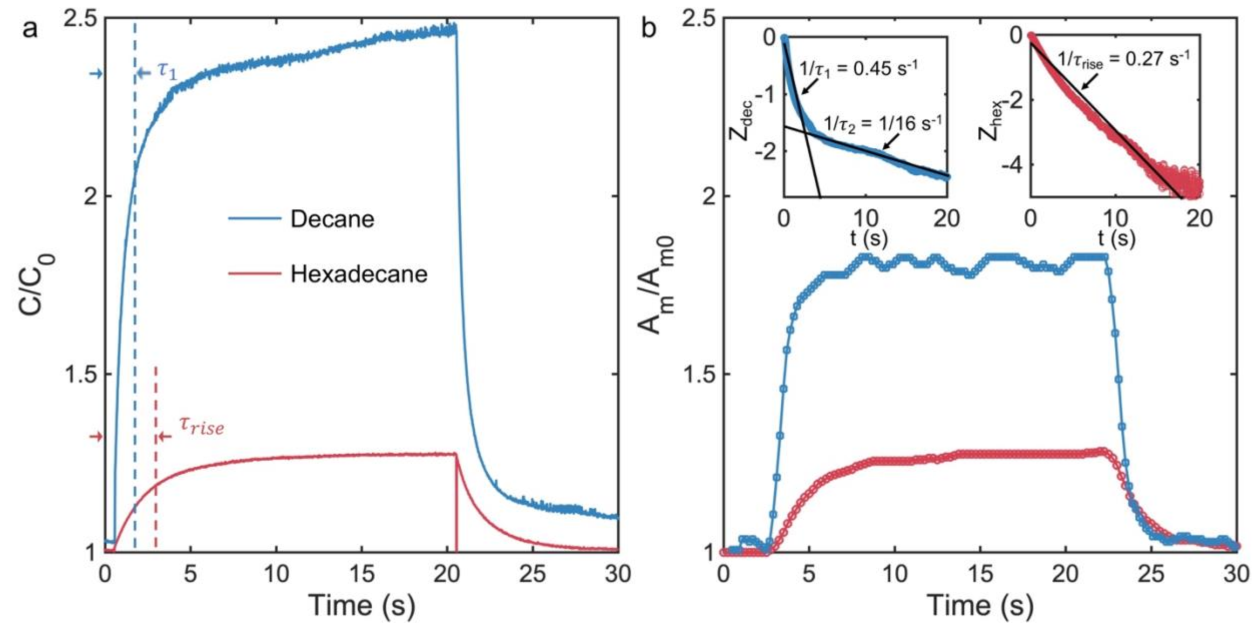

Supplementary Figure 7. Responses to stepwise changes in transmembrane potential. We simultaneously recorded capacitive current and bilayer images in response to stepwise changes in transmembrane voltage from 0 to 150 mV (0.025 Hz, 50% duty cycle). From the current data, we calculated nominal capacitance. Likewise, from bilayer images we computed bilayer area versus time. We found that while the increase in membrane area accounts for 99.5% of the change in capacitance for a DPhPC bilayer in hexadecane (**a**), both an increase in membrane radius (~75%) and a decrease in thickness (~28%) were needed to achieve the 150% increase in nominal capacitance for a bilayer in decane (**b**). The insets in (**b**) show the log-incomplete response values ($Z\ (t)$) as a function of time. The values for calculated using the function $Z(t) = ln\left(\frac{C(t)-C_{SS}}{C_0-C_{SS}}\right) = -\frac{1}{\tau}$, where *C(t)* is the membrane capacitance as a function of time, C_{ss} is the steady state membrane capacitance, and C_0 is the membrane capacitance at zero input voltage.

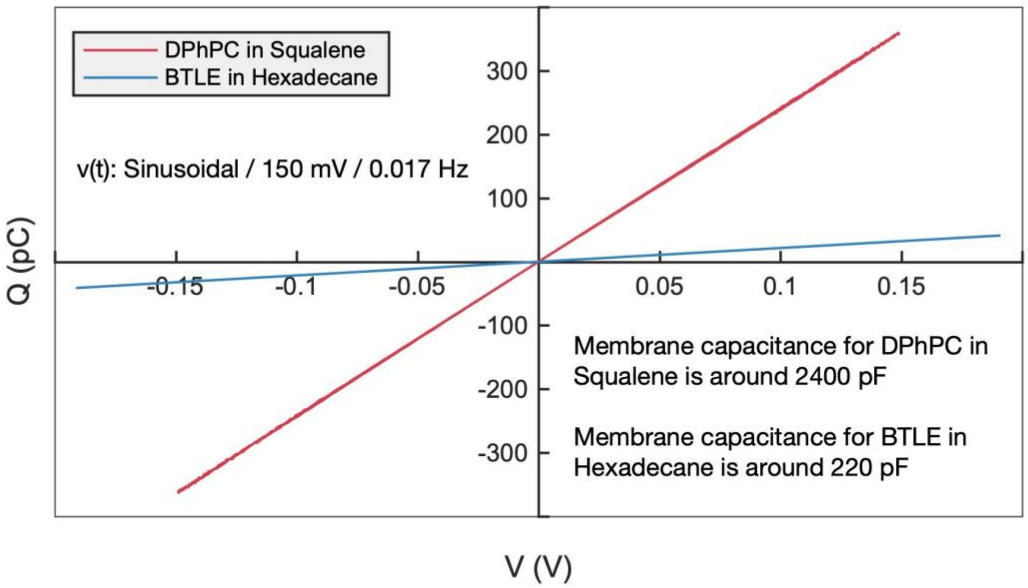

Supplementary Figure 8. Non-hysteretic *Q*-*v* for oil-free membranes. As discussed in the manuscript, *Q*-*v* hysteresis stems from the dynamics of EW and EC on the lipid membrane. These mechanisms are stronger when more oil is present in the bilayer. The linear *Q*-*v* data in this figure confirm these dependencies by showing the opposite, that EW and EC-induced *Q*-*v* hysteresis vanish when the membrane is completely oil-free. For example, a DPhPC lipid membrane formed in squalene produces an oil-free membrane that exhibits a linear (constant capacitance) and non-hysteretic, *Q*-*v* relationship (red line) at the same frequency for which memcapacitance exists for DPhPC bilayer in hexadecane or decane. Turning off the effects of EW and EC can also be achieved by using natural brain total lipid extracts (BTLE) to form a fixed-capacitance bilayer in hexadecane (blue line). This occurs because the BTLE membrane exists in a solvent-free state[10] at room temperature, which is far below the phase temperature of BTLE lipids (>35°C). Note, that oil-free bilayers also correspond to larger interfacial areas, which explains the higher net capacitance and charge observed for these bilayers in the same voltage range.

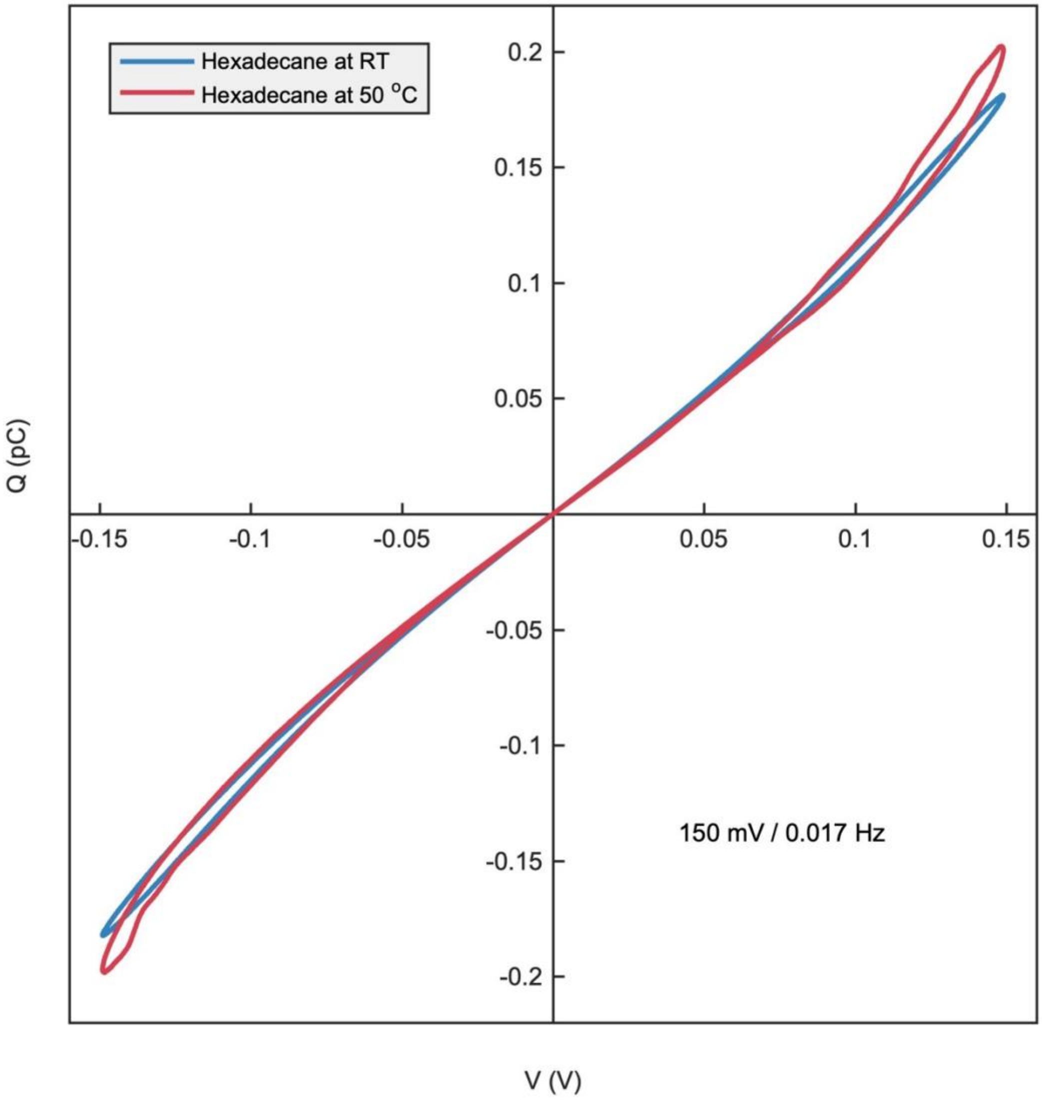

Supplementary Figure 9. Pinched hysteresis and dynamics response of the hexadecane system at 50 °C. The results show that the decreased viscosity of the oil at a higher temperature leads to a more pronounced hysteresis and nonlinearity.

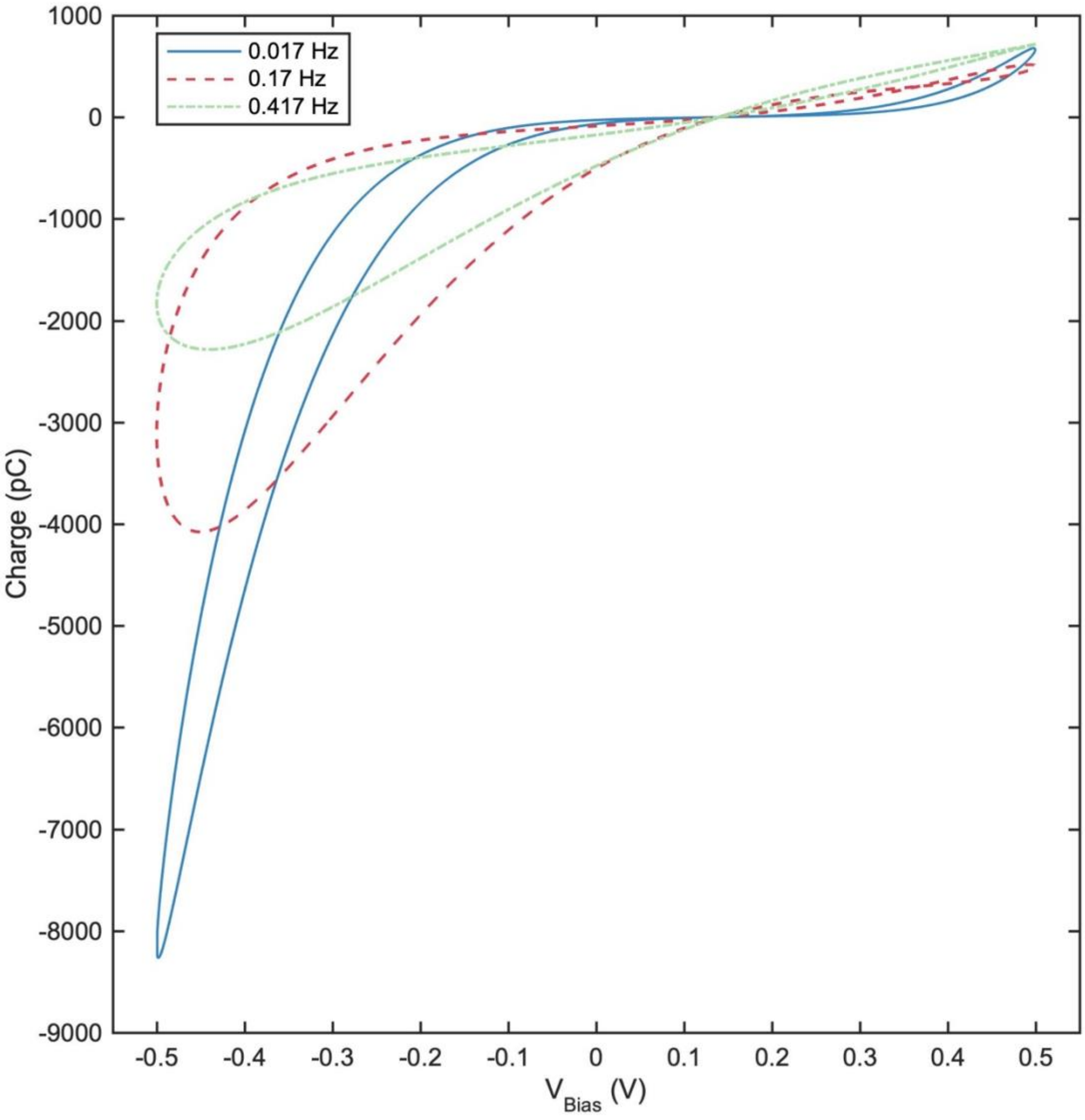

Supplementary Figure 10. Simulated charge versus voltage responses for an asymmetric DPhPC:DOPhPC membrane in decane. We used Eqs. 3 and 4 with damping and stiffness parameters from Table 1 to simulate the charge versus voltage response of an asymmetric membrane at a higher range of bias voltages (|V| = 500 mV). In this case, *v*(*t*) in the Eqs. 3 and 4 is replace by *v*(*t*)*-140* to account for the intrinsic membrane voltage created by leaflet asymmetry. The results show an asymmetric, pinched, hysteretic relationship in the *Q*-*v* plane. Note that, unlike

the results displayed in Figure 4, Q-v relationship displays a larger hysteresis lobe in response to positive voltages.

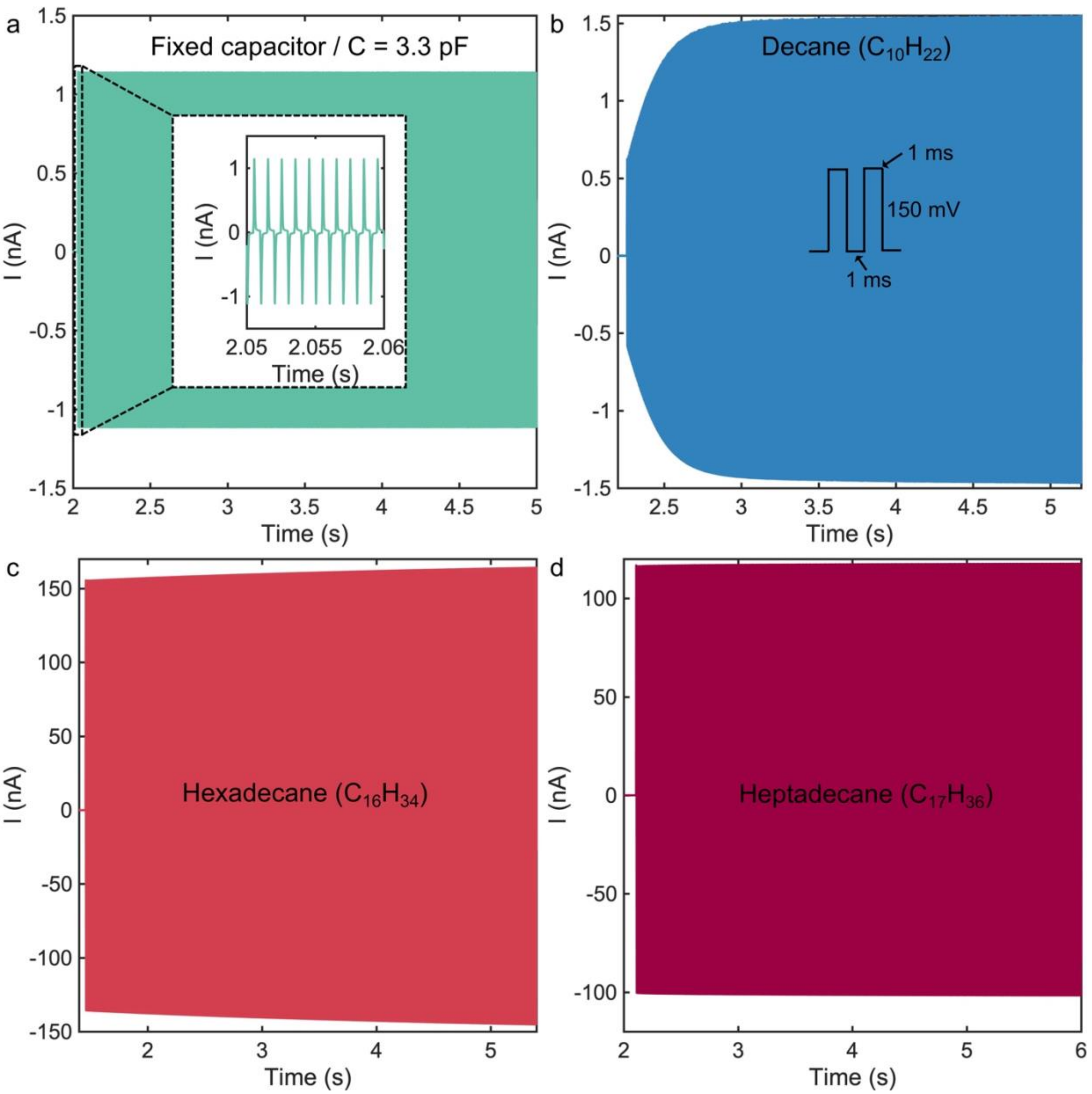

Supplementary Figure 11. Capacitive current responses to a train of voltage pulses. a, For a fixed 3.3pF solid-state capacitor, peak capacitive current, I_{peak}, is constant in response to a train of pulses. This data serves as a control experiment. **b** and **c**, The dynamic responses of I_{peak} for a symmetric DPhPC membrane in decane and hexadecane, respectively. The results show an accumulated increase in I_{peak}—highlighting the short-term plasticity of the systems. **d**, A membrane-based control case showing the I_{peak} response of a symmetric, oil-free DPhPC

membrane in heptadecane ($C_{17}H_{36}$). Analogous to the results in Supplementary Figure 8, these data show no significant changes in I_{peak}, due to the fact that this oil does not remain in the membrane (i.e., the bilayer formed is solvent-free), which minimizes both EW and EC effects on bilayer geometry.

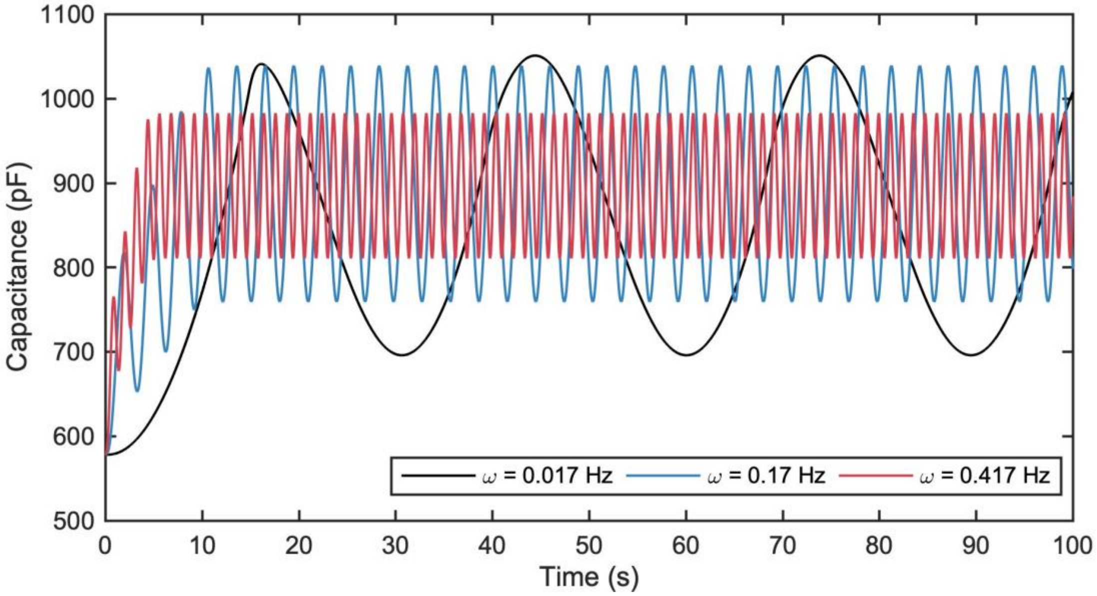

Supplemental Figure 12. Simulated lipid membrane capacitance in response to sinusoidal voltage waveforms. We simulate a DPhPC in decane in response to sinusoidal voltage and plotted the capacitance response of the lipid membrane with respect to time. The response matched well

(MAPE ranging between 0.26% to 3% and R^2 ~0.95) our experimental observation that the capacitance exhibited a transient response before reaching steady state at all frequencies.

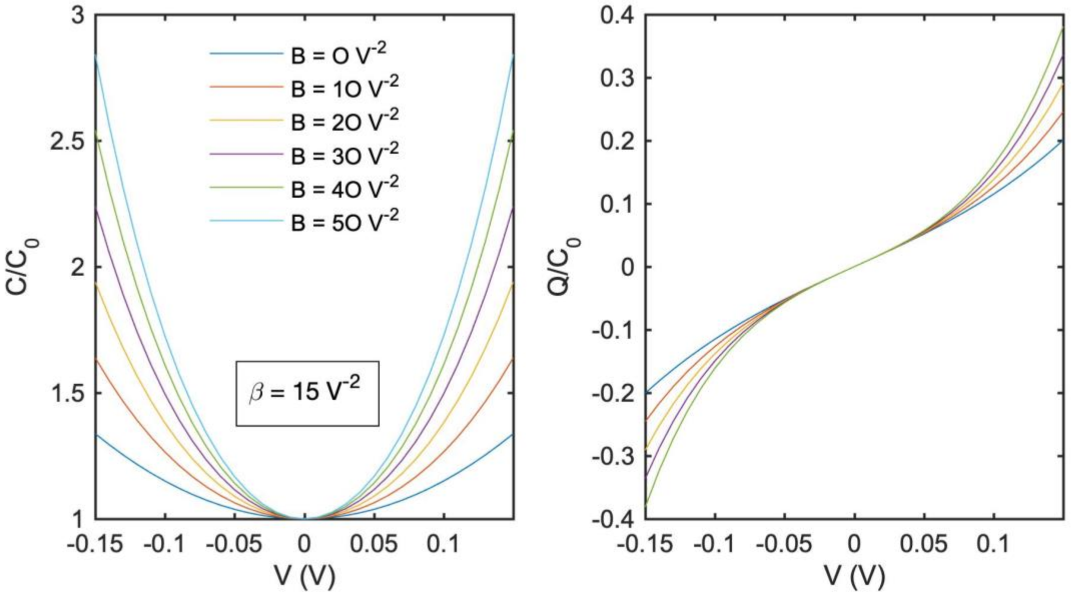

Supplemental Figure 13. Highlighting the impact of EW and EC coupling on nonlinearity of the system. Using Eq. S.8 we fixed the value of β to 15 and varied the B from 0 to 50 to highlight the impact of EW and EC coupling on the nonlinearity of the system. Both the C-v and Q-v responses become significantly more nonlinear as B increased.

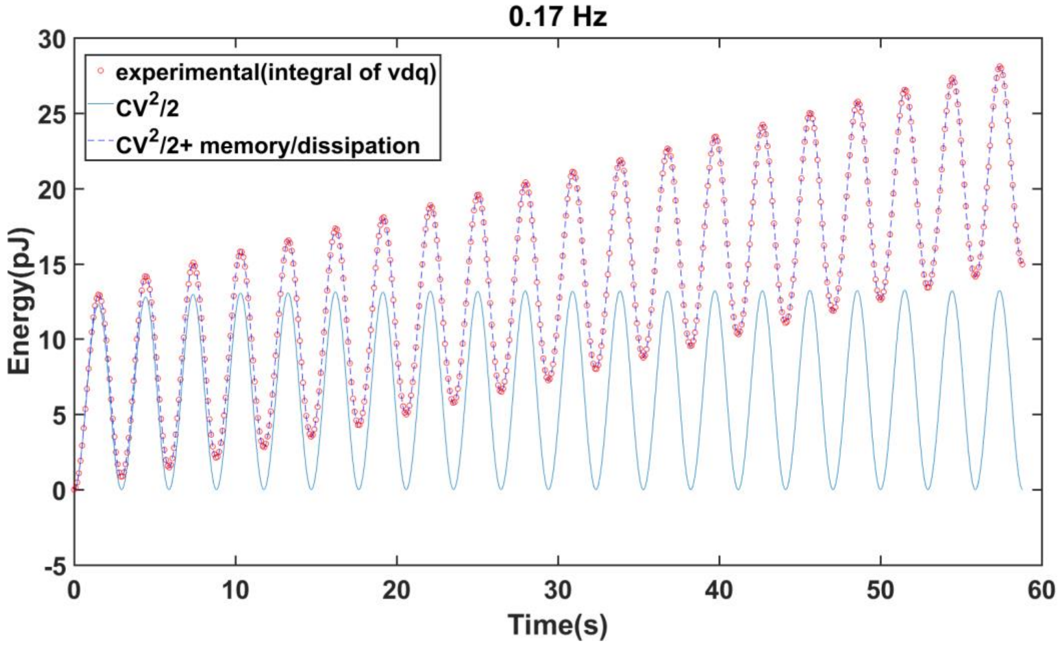

Supplementary Figure 14. Energy dissipation by a DPhPC bilayer in decane. The results show that the energy calculated using Eq. S.19 matched exactly the experimentally determined energy in the system when subjected to a 0.017 Hz sine wave. We obtained the red curve by integrating the product of the current and voltage with respect to time ($dQ = idt$).

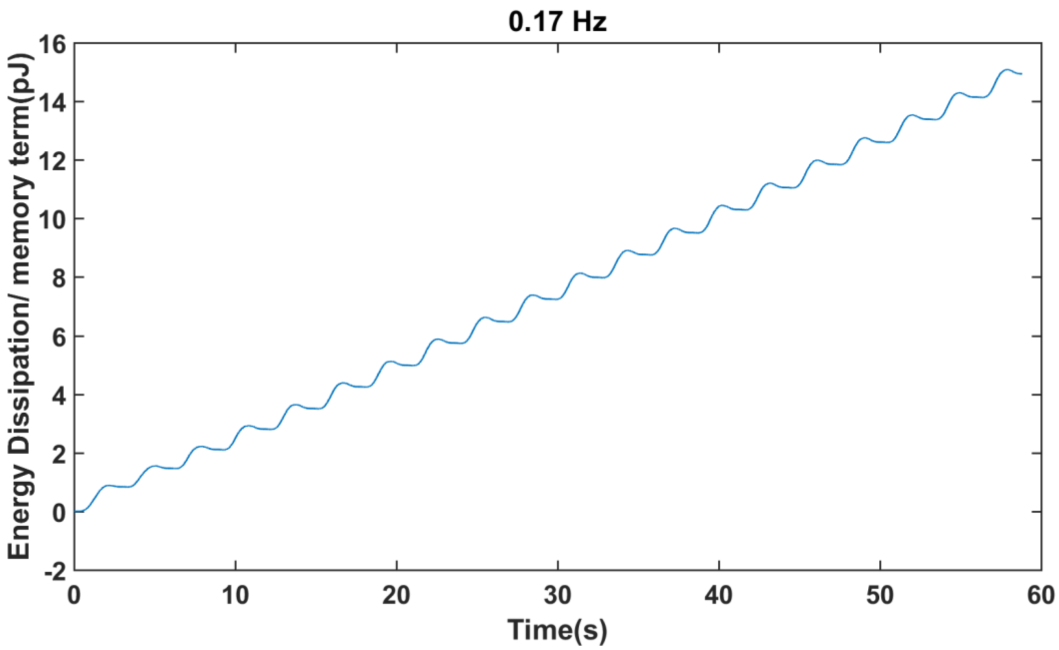

Supplementary Figure 15. Simulated memory/dissipation energy term with respect to time of a decane-based device. Energy is required to drive the mechanical/geometrical changes in our capacitive interface, which is dissipated as heat into the system.

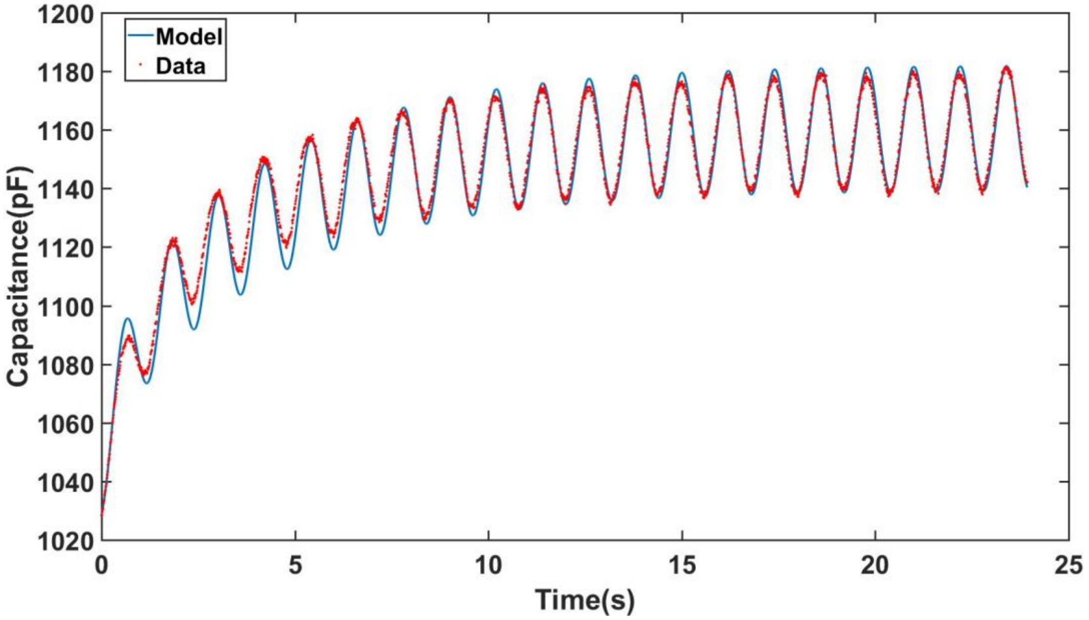

Supplementary Figure 16. Experimental and simulated data for a DPhPC bilayer in hexadecane excited at 0.417 Hz. The model, using fitted parameters from Table S1, successfully captures the transient and steady-state dynamic changes in capacitance in response to a sinusoidal voltage.

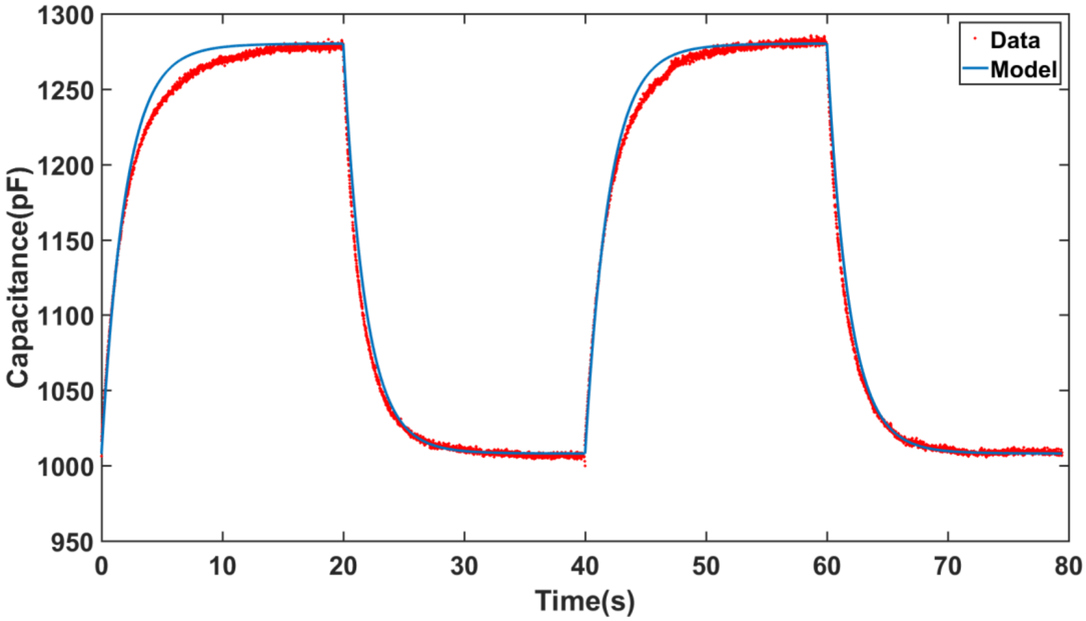

Supplementary Figure 17. Experimental and simulated data for a DPhPC bilayer in hexadecane in response to stepwise voltage changes. We used the damping and stiffness parameters from Table S1 to simulate the response of a hexadecane system to a stepwise increase in voltage from 0 mV to 150 mV.

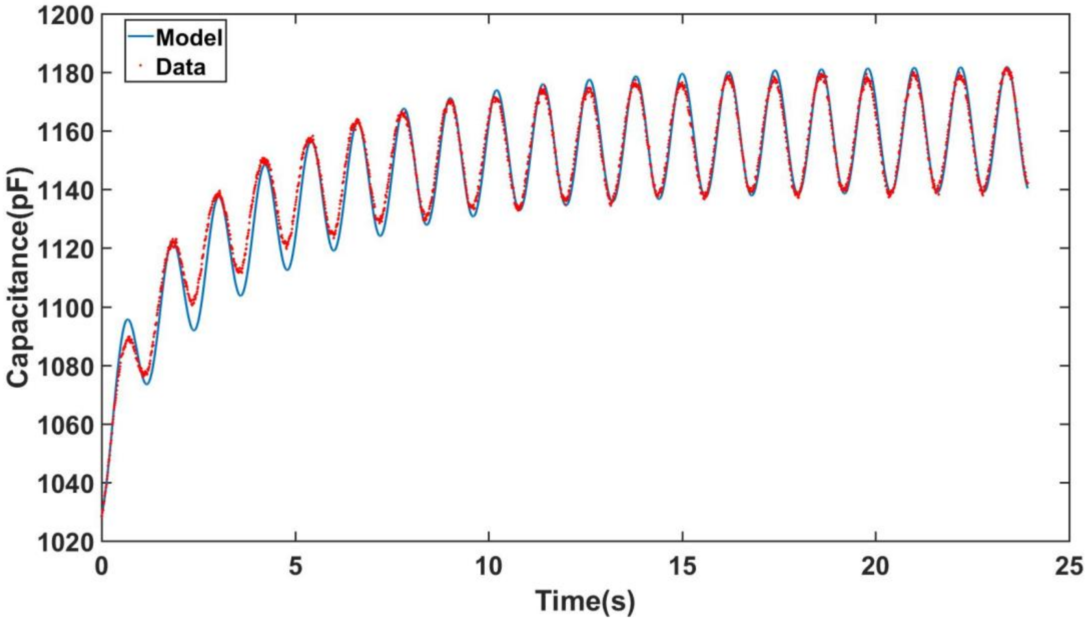

Supplementary Figure 18. Experimental and simulated data for a DPhPC bilayer in decane excited at 0.417 Hz. The model successfully captures the dynamic changes in capacitance in response to a sinusoidal voltage.

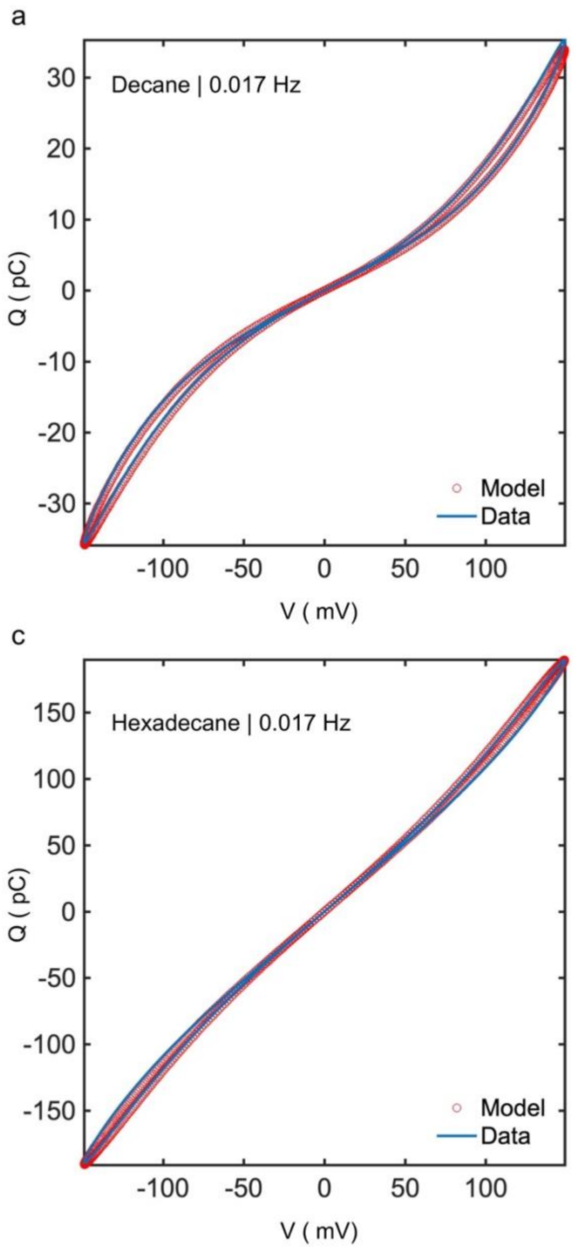

Supplementary Figure 19. Simulated versus experimental pinched, hysteresis *Q*-*v* relationships. We show steady-state pinched-hysteresis response at two different frequencies (0.017 Hz and 0.17 Hz) for decane (**a** and **b**) and hexadecane (**c** and **d**).

Supplementary References

1 Cooper, G. M. & Hausman, R. E. *The cell: Molecular approach*. (Medicinska naklada, 2004).

2 Singer, S. J. & Nicolson, G. L. The fluid mosaic model of the structure of cell membranes. *Science* **175**, 720-731 (1972).

3 White, S. H. & Thompson, T. Capacitance, area, and thickness variations in thin lipid films. *Biochimica et Biophysica Acta (BBA)-Biomembranes* **323**, 7-22 (1973).

4 Taylor, G. J., Venkatesan, G. A., Collier, C. P. & Sarles, S. A. Direct in situ measurement of specific capacitance, monolayer tension, and bilayer tension in a droplet interface bilayer. *Soft Matter* **11**, 7592-7605 (2015).

5 Venkatesan, G. A. *et al.* Adsorption kinetics dictate monolayer self-assembly for both lipid-in and lipid-out approaches to droplet interface bilayer formation. *Langmuir* **31**, 12883-12893 (2015).

6 Tamaddoni, N., Taylor, G., Hepburn, T., Michael Kilbey, S. & Sarles, S. A. Reversible, voltage-activated formation of biomimetic membranes between triblock copolymer-coated aqueous droplets in good solvents. *Soft Matter* **12**, 5096 - 5109, doi:10.1039/C6SM00400H (2016).

7 Holden, M. A., Needham, D. & Bayley, H. Functional Bionetworks from Nanoliter Water Droplets. *J. Am. Chem. Soc.* **129**, 8650-8655 (2007).

8 Najem, J. S. *et al.* Memristive Ion Channel-Doped Biomembranes as Synaptic Mimics. *ACS Nano* **12**, 4702-4711, doi:10.1021/acsnano.8b01282 (2018).

9 Gross, L. C. M., Heron, A. J., Baca, S. C. & Wallace, M. I. Determining Membrane Capacitance by Dynamic Control of Droplet Interface Bilayer Area. *Langmuir* **27**, 14335-14342, doi:10.1021/la203081v (2011).

10 Taylor, G. J., Heberle, F. A., Katsaras, J., Collier, C. P. & Sarles, S. A. Capacitive Detection of Low-Enthalpy, Higher-Order Phase Transitions in Synthetic and Natural Lipid Membranes. *Biophysical Journal* **114**, 551a-552a, doi:https://doi.org/10.1016/j.bpj.2017.11.3012 (2018).
