# Supplementary material for: Dynamical nonlinear memory capacitance in biomimetic membranes
Source: Nat Commun. 2019 Jul 19;10:3239. doi: 10.1038/s41467-019-11223-8 (PMC6642212; doi:10.1038/s41467-019-11223-8)
Supplement: Supplementary file 3 — Description of Additional Supplementary Files [file 41467_2019_11223_MOESM3_ESM.pdf]

Description of Additional Supplementary Files

Supplementary Movie 1:

Assembly and formation of a DPhPC lipid bilayer in Decane oil

Supplementary Movie 2:

Assembly and formation of a DPhPC lipid bilayer in Hexadecane oil

Supplementary Movie 3:

Assembly and formation of a DPhPC lipid bilayer in Squalene oil

Supplementary Movie 4:

Geometrical deformation of a DPhPC membrane in Hexadecane in response to a sinusoidal voltage waveform

Supplementary Movie 5:

Geometrical deformation of a DPhPC membrane in Decane in response to a sinusoidal voltage waveform
